# Urine proteomics identifies biomarkers for diabetic kidney disease at different stages

**DOI:** 10.1186/s12014-021-09338-6

**Published:** 2021-12-29

**Authors:** Guanjie Fan, Tongqing Gong, Yuping Lin, Jianping Wang, Lu Sun, Hua Wei, Xing Yang, Zhenjie Liu, Xinliang Li, Ling Zhao, Lan Song, Jiali He, Haibo Liu, Xiuming Li, Lifeng Liu, Anxiang Li, Qiyun Lu, Dongyin Zou, Jianxuan Wen, Yaqing Xia, Liyan Wu, Haoyue Huang, Yuan Zhang, Wenwen Xie, Jinzhu Huang, Lulu Luo, Lulu Wu, Liu He, Qingshun Liang, Qubo Chen, Guowei Chen, Mingze Bai, Jun Qin, Xiaotian Ni, Xianyu Tang, Yi Wang

**Affiliations:** 1 The Second Affiliated Hospital of Guangzhou University of Chinese Medicine, Guangzhou, 510120 China; 2 The Second Clinical College of Guangzhou, University of Chinese Medicine, Guangzhou, 510120 China; 3 Guangdong Provincial Hospital of Chinese Medicine, Guangzhou, 510120 China; 4 Guangdong Provincial Academy of Chinese Medical Sciences, Guangzhou, 510120 China; 5 Beijing Pineal Health Management Co., Ltd, Beijing, 102206 China; 6 State Key Laboratory of Proteomics, National Center for Protein Sciences, Beijing Proteome Research Center, Institute of Lifeomics, Beijing, 102206 China; 7 Chongqing Key Laboratory of Big Data for Bio Intelligence, School of Bioinformation, Chongqing University of Posts and Telecommunications, Chongqing, 400065 China

**Keywords:** Urine, Proteomics, DKD, Progression monitoring

## Abstract

**Background:**

Type 2 diabetic kidney disease is the most common cause of chronic kidney diseases (CKD) and end-stage renal diseases (ESRD). Although kidney biopsy is considered as the ‘gold standard’ for diabetic kidney disease (DKD) diagnosis, it is an invasive procedure, and the diagnosis can be influenced by sampling bias and personal judgement. It is desirable to establish a non-invasive procedure that can complement kidney biopsy in diagnosis and tracking the DKD progress.

**Methods:**

In this cross-sectional study, we collected 252 urine samples, including 134 uncomplicated diabetes, 65 DKD, 40 CKD without diabetes and 13 follow-up diabetic samples, and analyzed the urine proteomes with liquid chromatography coupled with tandem mass spectrometry (LC–MS/MS). We built logistic regression models to distinguish uncomplicated diabetes, DKD and other CKDs.

**Results:**

We quantified 559 ± 202 gene products (GPs) (Mean ± SD) on a single sample and 2946 GPs in total. Based on logistic regression models, DKD patients could be differentiated from the uncomplicated diabetic patients with 2 urinary proteins (AUC = 0.928), and the stage 3 (DKD3) and stage 4 (DKD4) DKD patients with 3 urinary proteins (AUC = 0.949). These results were validated in an independent dataset. Finally, a 4-protein classifier identified putative pre-DKD3 patients, who showed DKD3 proteomic features but were not diagnosed by clinical standards. Follow-up studies on 11 patients indicated that 2 putative pre-DKD patients have progressed to DKD3.

**Conclusions:**

Our study demonstrated the potential for urinary proteomics as a noninvasive method for DKD diagnosis and identifying high-risk patients for progression monitoring.

**Supplementary Information:**

The online version contains supplementary material available at 10.1186/s12014-021-09338-6.

## Background

In 2019, more than 463 million people worldwide were estimated to be living with diabetes, representing 9.3% of the global adult population (20–79 years) [1]. Among these people, 20% to 40% will progress to DKD [2], which remains a leading cause of morbidity and mortality in people with type 2 diabetes [3–5]. DKD patients are at significant risk of progression to ESRD and cardiovascular diseases. Therefore, early detection, prevention, and treatment are of great importance for disease management [6]. Clinically, the diagnosis of DKD is based on the measurement of eGFR, albuminuria and urinary microalbumin creatinine ratio (UACR) along with other clinical features [7]. The “gold standard” for definitive diagnosis of DKD requires kidney biopsy with which an expert renal pathologist makes the diagnosis from histological tissue. However, the procedure is invasive and sometimes dangerous, and the evaluation process can be biased by human judgment [8]. It is important to find a noninvasive test method that can complement or replace renal puncture.

DKD is divided into five stages according to clinical guidelines [9, 10]. The stage 1 (DKD1) and stage 2 (DKD2) DKDs are preclinical stages, and are characterized by an increase in glomerular filtration rate (GFR), normal albuminuria or intermittent microalbuminuria. DKD3, characterized by persistent microalbuminuria, mild hypertension, and a normal or slight decline in GFR, is the onset of clinical stage [11]. Patients with DKD4 exhibit clinical symptoms of edema and hypertension, along with an increase in albuminuria, which is difficult to treat [12]. In the overt DKD4, as the glomerular filtration rate (GFR) declines, the albumin-to-creatinine ratio (ACR) would further increase (> 300 mg/g). Under this circumstance, taking any medicine could increase the kidney burden thus exacerbate the disease. With proper clinical intervention, the DKD progression can often be delayed or even reversed before it progresses to stage 4. Therefore, monitoring the kidney function of DKD3 patients is critical to the medical treatments and delaying disease prognosis.

In the past years, several noninvasive methods have been proposed, mostly based on the ‘omics’ techniques for the evaluation of urine or serum biomarkers [13, 14]. Among these approaches, urinary proteome analysis has gained popularity, and has the potential to be transitioned towards clinical implementation [15, 16]. Measuring urine proteome is non-invasive, and quantitative determining the urinary proteins and peptides can be an enabling platform to distinguish disease stages or monitor response to therapies. Many efforts have been put into finding biomarkers for CKD, aiding risk assessment for DKD patients. Using high-resolution capillary electrophoresis coupled with electrospray-ionization mass spectrometry, a panel of 40 biomarkers were identified from the urinary peptides and they were reported to be able to differentiate healthy individuals from diabetic patients with persistent normal albuminuria, low-grade albuminuria, or nephropathy (PREDICTION) [17]. Furthermore, it could distinguish patients with DKD from patients with other CKDs. However, the exact protein identities of these peptide biomarkers were not clear. A metabolic study utilized normal phase liquid chromatography coupled with time-of-flight mass spectrometry (NPLC-TOF/MS) investigated the profile of the plasma phospholipids of type 2 diabetes and DKD [18] and found that 2 novel biomarkers, PI C18:0/22:6 and SM dC18:0/20:2, could be used to distinguish healthy individuals, type 2 diabetes cases and DKD cases from each other, but these two biomarkers cannot distinguish the stages of DKD.

A CKD273 classifier was successful in using urine peptides to predict the development of DKD before patients developed microalbuminuria [19]. A work by Liao, W.-L. et al. identified haptoglobin (HPT) and α-1-microglobulin/bikunin precursor (AMBP) as two biomarkers with the highest ability to distinguish between healthy individuals and patients with nephropathy, and between diabetic patients with or without DKD [20] in a Taiwanese population. However, the study population was limited to patients with an ACR of less than 300 mg/g, and none of the patients had stage 3 of CKD. In addition, the information on the use of insulin or other drugs (such as renin-angiotensin system antagonists) that may alter renal function was not provided.

Currently, clinical DKD surveillance relies on the measurements of eGFR and UACR, along with other physical and clinical parameters. The accuracy of these tests is not ideal and often depends on the results of multiple tests [21]. Here, we report a streamlined urine proteomics workflow to monitor DKD at different stages using liquid chromatography coupled with tandem mass spectrometry (LC–MS/MS). Urine proteomes of diabetes without complications, DKD, and other nephropathy patients without diabetes were measured and used to build logistic regression models to differentiate DKD from uncomplicated diabetes and different stages of DKD.

## Materials and methods

### Sample collection

A total of 239 urine samples from 236 patients, including diabetes without nephropathy (n = 134), diabetic kidney disease (n = 65), and nephropathy without diabetes (n = 40), were collected in Guangdong Provincial Hospital of Chinese Medicine, Guangzhou, China. The batch 1 dataset of 30 urine samples was collected in year 2018, including 21 cases of diabetes without nephropathy and 9 cases of DKD; the batch 2 dataset of 209 urine samples was collected in year 2019, including diabetes without nephropathy (n = 113), DKD (n = 56) and nephropathy without diabetes (n = 40). DKDs were separated into two stages as DKD3 (Urinary Albumin/Creatinine Ratio: 30–300 mg/g) and DKD4 (Urinary Albumin/Creatinine Ratio > 300 mg/g).

### Sample preparation and nano HPLC–MS analysis

Midstream of the first-morning urine was obtained and stored at − 80 °C. One milliliter of all urine samples was centrifuged at 176,000 g for 1 h and the pellet was collected. The pellet was resuspended with 160 μl of resuspension buffer (100 mM Tris, pH 7.4) with 50 mM dithiothreitol (DTT). The suspension was then heated at 65 °C for 30 min and centrifuged at 176,000 g for 0.5 h. The pellet was resuspended with 30 μl NH_4_HCO_3,_ heated at 95 °C for 3 min and cooled to room temperature, then digested by trypsin or 12 h.

The digested peptides were vacuum dried and re-dissolved in 0.1% formic acid and resolved on an UltiMate 3000 RSLCnano System (Thermo Fisher Scientific) operating on a 20 min linear gradient for batch 1 and 30 min for batch 2 (5–35% acetonitrile in 0.1% formic acid) at a flow rate of 600 nl/min. Tandem mass spectra were acquired on a QExactive HF mass spectrometer (Thermo Fisher Scientific) in the data-dependent mode. Quality control samples were made prepared from trypsin digests of 293 T cells and were routinely analyzed to assess the LC–MS/MS sensitivity and reproducibility.

### Protein identification and label-free quantification

MS data were processed on the Firmiana platform [22]. Proteins were identified against the NCBI human RefSeq protein database (released on 04/07/2013, 32,015 entries) using the MASCOT search engine (Matrix Science, version 2.3.01). Mass tolerance was set as 20 ppm for precursor ions and 0.05 Da for product-ions, respectively. Up to one missed cleavage was allowed for trypsin digestion. Cysteine carbamidomethylation was considered as a fixed modification, N-terminal acetylation and methionine oxidation were considered as dynamic modifications. 1% FDR on both the peptide and protein levels estimated by searching a decoy database were allowed. Only identifications with ≥ 1 unique and strict peptides and ≥ 2 strict peptides (ion score > 20) or ≥ 3 strict peptides, which was comparable to 1% FDR at the protein level, were used for subsequent analyses. For protein quantification, intensity based absolute quantification (iBAQ) algorithm [23] was used. To normalize the differences in sample amounts, iBAQ values were converted to iFOTs (fraction of total) calculated by dividing the iBAQ value of each protein by the total iBAQ of the sample followed by multiplying 10^5^ for easy visualization. All missing values were substituted with zero.

### Statistical analysis

Pathway enrichment analysis was performed using reactome (https://reactome.org/) [24]. Principal component analysis (PCA) was performed by the sklearn (0.21.2) package with Python on validation dataset. Two components were used for data visualization. Protein differential excretion was assessed with Kruskal–Wallis test and Mann–Whitney U test. The correlation analyses between clinical and proteomics data and the statistical analyses were calculated by scipy (version 1.3.0) package with Python. For high-dimensional MS data, dimension reduction was applied to avoid over-fitting. We first defined the differentially expressed proteins as the candidate biomarker set, then go through the potential combinations of less than or equal to 4 proteins. At last, a logistic regression model was carried out to select the optimal panel of biomarker.

A Logistic Regression Classifier (sklearn (0.21.2) package with Python) was built using batch1 data as training set and batch2 data as validation set. The performance of the model was evaluated by sensitivity and specificity computed based on the confusion matrix. The Receiver Operating Characteristic curve plotted sensitivity (True Positive Ratio) as the x axis and 1-specificity (False Positive Ratio) as the y axis. Feature selection was applied to select most important features that can make the model achieve a higher AUC (area under ROC curve). In Classification model 3, DKD3 progression risk was assessed by a risk score (ranging from 0 to 1), calculated by the predict_proba function in the sklearn (version 0.21.2) package based on Logistic Regression Classifier.

## Results

### Urine proteomics of uncomplicated diabetes without nephropathy, DKD, and CKD without diabetes

**Fig. 1:**
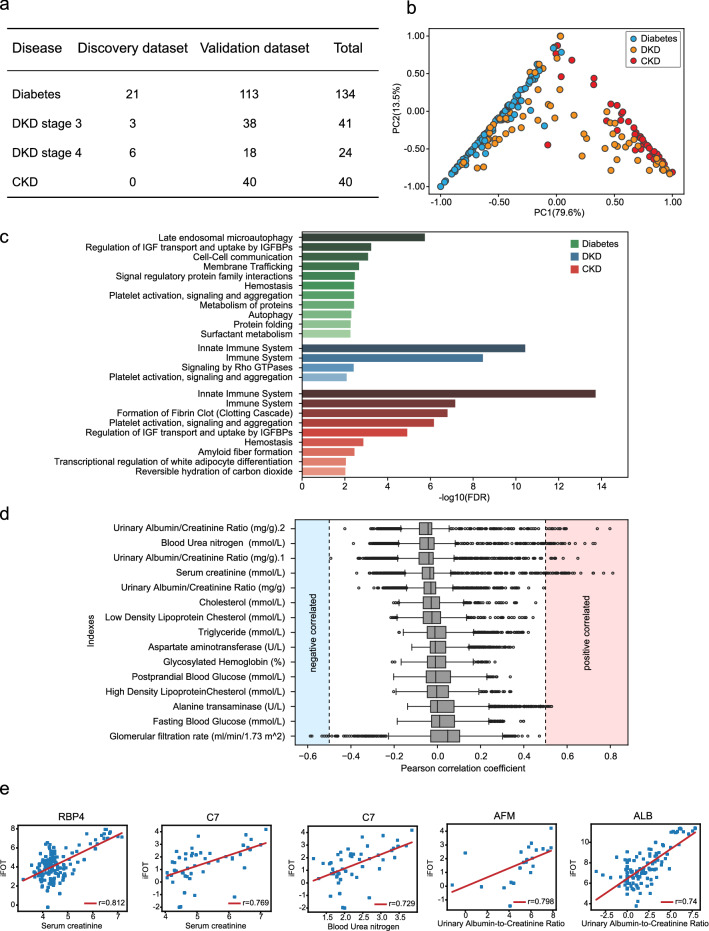
Urine proteomic analysis of Diabetes, DKD, and CKD. **a** Clinical stages and the number of samples used in the discovery and validation datasets. **b** Principal component analysis of the proteomics data. Each dot represents an urinary sample. Blue: Diabetes, Yellow: DKD, Red: CKD. **c** Reactome pathway analysis of the disease-specific DEPs (323 DEPs for diabetes, 98 DEPs for DKD, 88 DEPs for CKD). **d** Pearson correlation coefficients between the abundance of the 2,946 urine proteins and the 13 routinely tested clinical or health indexes. **e.** Scatter plots of selected high abundance urine proteins and kidney function indexes with strong positive correlations

We employed a previously published procedure for measuring urine proteomes [25] with minor modifications. This streamlined workflow achieved high efficiency and batch-to-batch reproducibility. The stability and reproducibility of the MS platform was ensured by running 293 T cell protein extracts as quality control (QC) samples during the data-collection period. The median of the Pearson correlation coefficient between QC samples was 0.93 (Additional file 1: Fig. S1a). With this procedure, we measured 239 urine samples from 236 patients, including uncomplicated diabetes without nephropathy (diabetes herein for simplicity, 134 samples from 132 patients), DKD (65 samples from 64 patients), and chronic kidney disease without diabetes (CKD herein for simplicity, 40 samples from 40 patients) (Fig. 1a, Additional file 3: Table S1). A total of 2946 proteins was identified and quantified with high analytical confidence (Additional file 1: Fig. s1b, Additional file 3: Table S1) with a median of 571 proteins identified per urine sample (Additional file 1: Fig. S1c).

Principle component analysis (PCA) of all proteins in the dataset showed that, when all samples were projected onto the first two principal components, representing 79.6% and 13.5% of the variance, respectively (Fig. 1b), diabetes and CKD can be clearly separated, whereas DKD appears to be in between. Next, we selected differentially excreted proteins (DEPs) in each group, using the following criteria: (1) the ratio of the mean of the highest group/second highest group >  = 2, and (2) the p-value of Kruskal–Wallis test < 0.05 (Additional file 4: Table S2). The 509 DEPs whose expressions were at least two-fold higher in one disease than in any other disease were defined as disease-specific DEPs (323 DEPs for diabetes, 98 DEPs for DKD, 88 DEPs for CKD). Reactome analysis showed that proteins specific in diabetes were enriched in late endosomal microautophagy, regulation of IGF transport and uptake by IGFBPs. In contrast, innate immune system and platelet activation were significantly up-regulated in both DKD and CKD, indicating that the two diseases share similar pathology. Moreover, enrichment of these pathways was higher in statistical significance between CKD and diabetes than those between DKD and diabetes. Notably, the formation of fibrin clot (clotting cascade) was up-regulated in CKD, but not in DKD (Fig. 1c).

To investigate whether the urinary proteome correlated with the clinical parameters assessed by serum tests, we performed correlation analysis for the 2946 proteins and 13 routinely tested clinical or health indexes. We calculated the Pearson correlation coefficient for each protein with each index (Fig. 1d, Additional file 4: Table S2). Strong correlations (Pearson correlation coefficient > 0.5 or < − 0.5) were found between 4 kidney functional indexes (serum creatinine, albumin-to-creatinine ratio, blood urea nitrogen, and glomerular filtration rate) and 46 urinary proteins. For example, serum creatinine was strongly correlated with the urinary protein abundance of RBP4 (retinol binding protein 4) and C7 (complement C7), with Pearson correlation coefficient of 0.812 and 0.769, respectively. C7 abundance was also correlated with blood urea nitrogen (Pearson correlation coefficient: 0.729) (Fig. 1e). Consistently, 3 proteins (ALB, RBP4, C7) were negatively correlated with glomerular filtration rate (Additional file 1: Fig. S1d). These results indicate that serum clinical indexes may be reflected in the urine proteome, which could provide additional biological insights into kidney dysfunctions in these diseases.

### Distinguish DKD from diabetes with a 2-protein Classifier

**Fig. 2:**
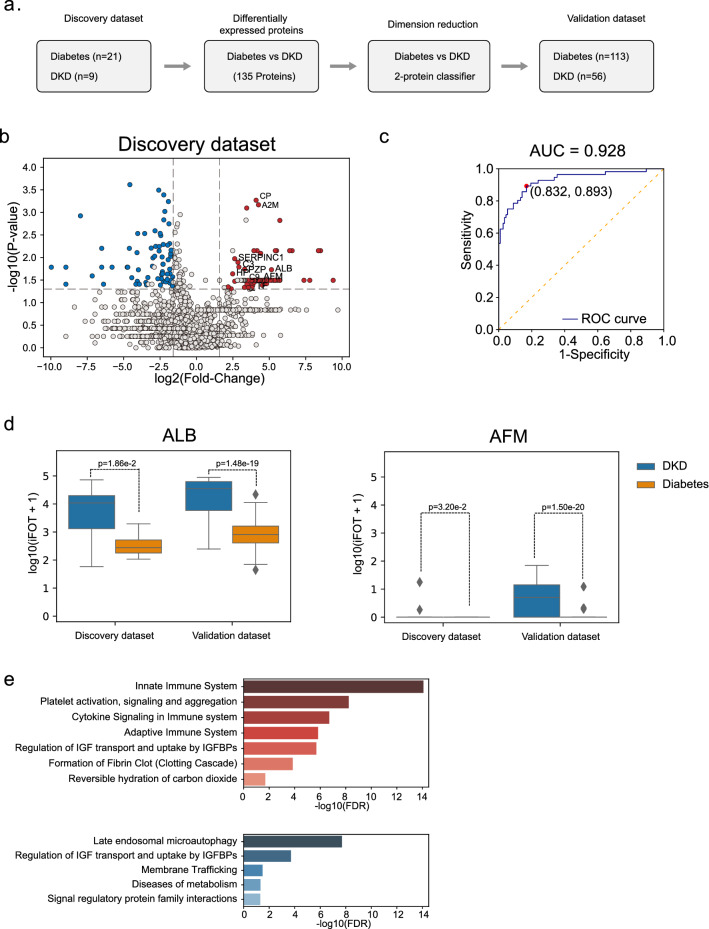
A classifier for distinguishing DKD from Diabetes.** a** A bioinformatic analysis workflow to find candidate biomarkers between the Diabetes and the DKD group. n: number of samples used in the analyses. **b** Volcano plot displaying the differentially expressed proteins between Diabetes and DKD. Red and Blue indicated proteins that were significantly enriched in DKD and Diabetes, respectively (p values < 0.05, more than threefold change). Other proteins were colored in grey. **c** ROC curve of distinguishing the Diabetes and DKD samples predicted by the 2-protein classifier. **d** Boxplots showing the ALB and AFM abundance in Diabetes and DKD in the two datasets (center line: median, bounds of box: 25th and 75th percentiles, and whiskers: from Q1-1.5*IQR to Q3 + 1.5*IQR, p-value calculated by Mann–Whitney U test). **e** Reactome pathway analysis of the up-regulated (red) and down-regulated (blue) DEPs in DKD samples

We used the dataset collected from year 2018 as the discovery dataset and the dataset collected from year 2019 as the validation dataset (Fig. 1a). To identify urinary biomarkers that distinguish DKD from diabetes, we determined DEPs between diabetes and the DKD group (including both DKD3 and DKD4 samples) (Fig. 2a). From the discovery dataset, which contained 21 diabetes and 9 DKD, we found 177 DEPs (Mann–Whitney U test p values < 0.05, fold-change of means > 3), among which 79 were up-regulated and 73 were down-regulated in DKD patients (Fig. 2b) Next, we selected high-abundant DEPs (mean iFOT > 1) in diabetes or DKD as potential biomarkers, resulting in 135 proteins (Additional file 5: Table S3). After dimension reduction, we showed that using 2 proteins (ALB, AFM) could readily distinguish DKD from diabetes (AUC: 0.928, accuracy: 85.2%, specificity: 83.2%, sensitivity: 87.5%, Fig. 2c) in the validation set. Both ALB and AFM were significantly excreted at higher levels in the validation dataset (Fig. 2d).

Reactome analysis revealed that the up-regulated DEPs were enriched in neutrophil degranulation, adaptive immune system, and complement cascade, while the down-regulated DEPs were enriched in membrane trafficking and cellular responses to stress (Fig. 2e). These results indicated the major functional differences between DKD and diabetics as revealed by the urine proteome. Close examination of the high abundance DEPs (top 50) revealed that liver- (29/50) and bone marrow (6/50)-specific proteins [26] were enriched in DKD, but low tissue specificity was found among those highly expressed in diabetes (Additional file 5: Table S3).

### Distinguish DKD3 from DKD4 with a 3-protein classifier

**Fig. 3:**
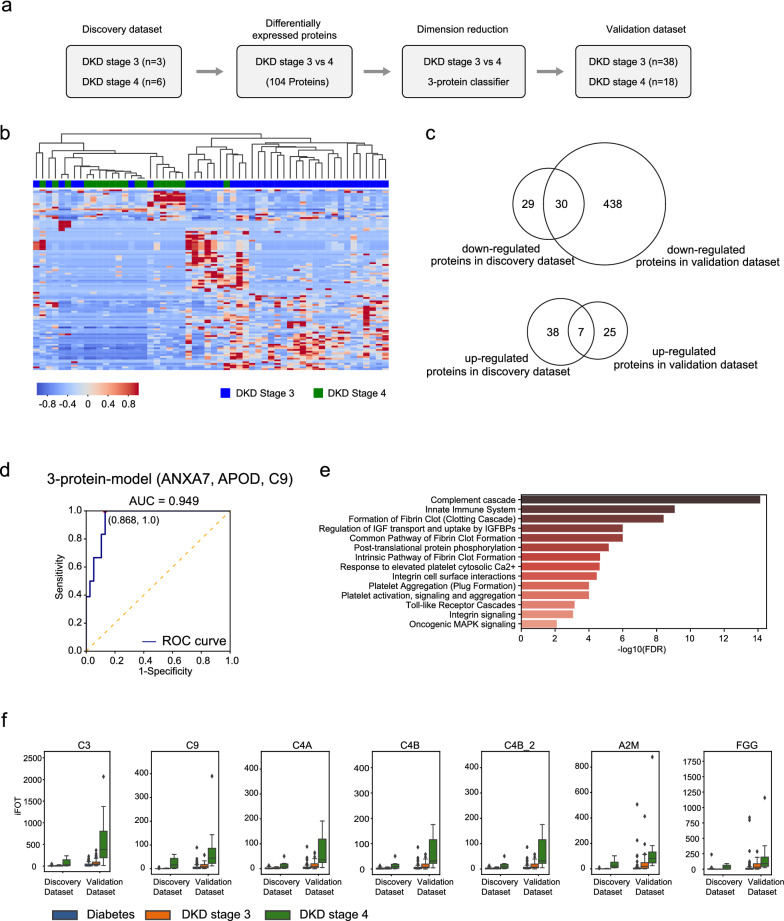
A classifier for distinguishing DKD4 from DKD3. **a** A bioinformatic analysis workflow to find candidate biomarkers between DKD3 and DKD4 samples. n: number of samples used in the analyses. **b** Hierarchical clustering of DKD3 and DKD4 DEPs using complete linkage. Protein expression values were normalized by z-scores. **c** Venn diagram indicating the overlap of DEPs between discovery and validation datasets. **d** ROC curve of distinguishing DKD3 and DKD4 predicted by a 3-protein classifier. **e** Reactome pathway analysis of the up-regulated DEPs in DKD4 samples. **f** Boxplots displaying the abundance of the complement component proteins at different stages of DKD and CKD in the two datasets

As DKD3 and DKD4 are managed differently in the clinics [27], a non-invasive test to distinguish the DKD stages could aid in disease management. To this end, we first used the DKD3 (n = 3) and DKD4 (n = 6) samples in the discovery set to find DEPs (Fig. 3a). Since the difference between DKD3 and DKD4 might be smaller than that between diabetes and DKD, we applied a more relaxed constraints for protein filtering (Mann–Whitney U test p < 0.05 or fold-change > 3 and detection frequency > 50%). We identified 104 DEPs with 45 up-regulated and 59 down-regulated proteins in DKD4 compared to DKD3 (Additional file 6: Table S4). An unsupervised hierarchical clustering analysis of the validation set using the 104 DEPs showed that DKD3 and DKD4 can be well separated (Fig. 3b), suggesting that significant differences have occurred when DKD3 progressed to DKD4. Using the same protein filtering criteria in the validation set, 32 up-regulated and 468 down-regulated proteins were found in DKD4 compared to DKD3 (Additional file 6: Table S4). A Venn diagram showed that 7 of the 45 (24.4%) up-regulated DEPs in the discovery set and 30 of the 59 (50.8%) down-regulated DEPs were also found in the validation set (Fig. 3c). After performing a dimension reduction, a 3-protein classifier could distinguish DKD3 from DKD4. Applying the prediction model to the validation set resulted in an ROC of 0.949 (accuracy: 85.7%, specificity: 86.8%, sensitivity: 83.3%, Fig. 3d). Reactome analysis revealed that the up-regulated DEPs in DKD3, including all 7 overlapping ones, were enriched in hemostasis and immune system, especially in complement cascade (Fig. 3e–f).

### Monitor the transition to DKD with a 4-protein classifier

**Fig. 4:**
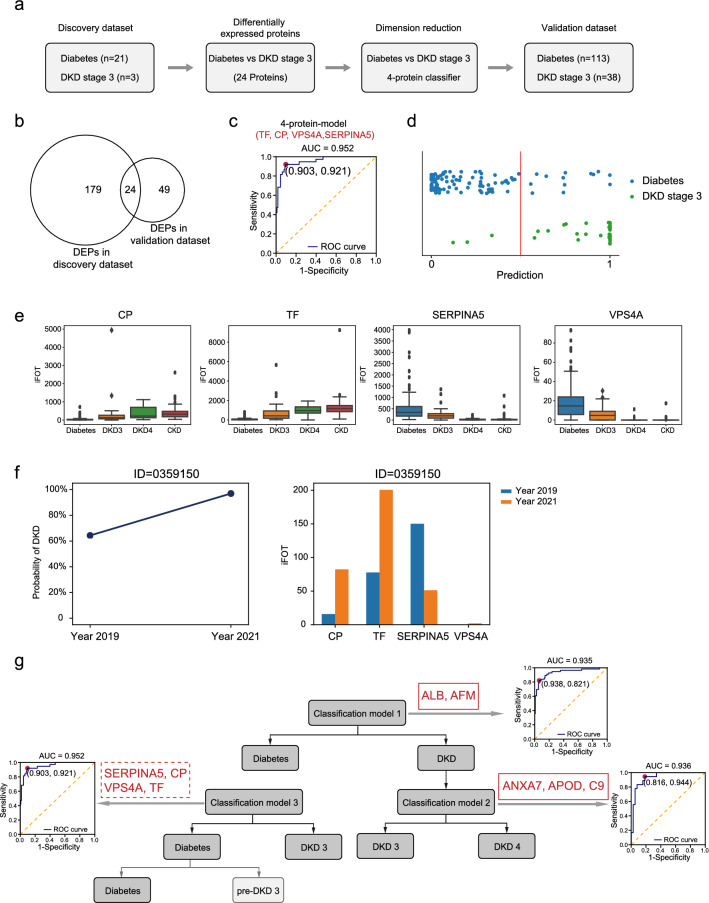
A classifier for monitoring early transition to DKD. **a** A bioinformatic analysis workflow to find candidate biomarkers between DKD3 and Diabetes samples. **b** Venn diagram showing the overlap of DEPs in the discovery and the validation set. **c** ROC curve of distinguishing DKD3 and Diabetes predicted by a 4-protein classifier. **d** Dotplot indicating the Diabetes and DKD samples and their predicted stages by the classifier. Each point represented one urine sample. Blue and green dots presents Diabetes and DKD, respectively. The x-axis indicates the predicted DKD stage 3. Blue dots positioned on the right side of the Prediction line were predicted incorrectly as DKD3 by the model and were used as putative pre-DKD in the subsequent analysis. **e** Boxplots displaying the iFOT intensities of the 4 biomarkers (CP, TF, SERPINA5, VPS4A) in Diabetes, DKD3, DKD4, and CKD samples. **f** The risk scores(left panel) and the 4-marker expression (right panel) of the patient (clinical ID: 8073510). **g **A schematic summary of bioinformatic analysis workflow that derived the 3 prediction models in this study

Since our goal is early diagnosis for DKD, we re-analyzed DKD3 and diabetes datasets to identify proteins that could implicate the transition from diabetes to DKD (Fig. 4a). As small sample size would result in poor estimation and low statistical power, in the discovery set, we performed DEP analysis between diabetes group (n = 21) and the DKD group (n = 3) with the following 2 criteria: (1) the mean iFOT in DKD group was 2 times higher than that in diabetes group; (2) detected in at least 2 samples in the DKD group, resulting in 203 DEPs (Additional File 7: Table S5). Next, we identified DEPs (Mann–Whitney U test p < 0.05, fold change > 2 and detection frequency > 50%) in the validation dataset, resulting in 73 DEPs (Additional File 7: Table S5). After performing the dimension reduction from the 24 overlapping DEPs from discovery and validation datasets, a 4-protein classifier (SERPINA5, VPS4A, CP, TF) could distinguish diabetes from DKD3 (Fig. 4b). Applying this classifier to the validation dataset resulted in an area under the ROC curve of 0.952 (accuracy: 89.4%, specificity: 88.5%, sensitivity: 92.1%, Fig. 4c) (see Additional file 7).

We noted that 13 of the 113 diabetic samples were classified as DKD3 (Fig. 4d). This seemingly false-positive result may indicate the inaccuracy of the classifier; alternatively, it could also indicate clinical misdiagnosis, as progression from diabetes to early stage of DKD (pre-DKD3) is difficult to detect. We then explored the possibility of inferring the uncomplicated diabetes to “pre-DKD3” progression based on the difference between diabetes and these 13 false-positive DKD3 samples. To this end, we identified DEPs among the 13 false-positive DKD3 and the rest of the diabetes samples in the validation set. Using the criteria described (Wilcoxon p value < 0.05, ratio of means >  = 2 or <  = 0.5, and frequency > 0.6), we found 35 DEPs (16 upregulated and 19 downregulated, Additional file 8: Table S6), which include all 4 classifier proteins, as expected. As shown in Fig. 4e, the levels of the 4 classifier proteins, as well as several other DEPs also showed significant changes at other stages of the disease, and the changes were more significant as the severity of the disease progress. Together, these data indicate that the dysfunctional pathways in different stages of the diseases share similarity and DKD is a progressive disease.

To test the predictive power of our model, we analyzed longitudinal urine samples collected from 11 diabetic patients, among which 6 were predicted as the high-risk “pre-DKD” patients. We assess the progression to DKD by calculating the risk scores for each urine sample using model 3. Four of the 6 pre-DKD patients were classified as DKD3 once again (Additional file 2: Fig. S2). Importantly, one of the patients (clinical ID: 0359150) was diagnosed as DKD3 by clinical index (Fig. 4f, Additional file 9: Table S7). Moreover, one patient (clinical ID: 8073510), who was classified as diabetes in the validation dataset and re-classified as DKD3 in 2020, was admitted to hospital in March 2021 for DKD treatment, and then recovered in July 2021 after Valsartan Capsules treatment. These results suggest urine proteomics have the potential to monitor DKD progression, especially for high-risk diabetes patients.

## Discussion

In this study, we conducted urinary proteomic studies on 236 patients. This streamlined, sedimentation-based method allowed us to detect 559 proteins/sample on average and a total of 2946 proteins, requiring 0.5 h/sample of processing time. The data allowed us to build models that could distinguish DKD from diabetes (AUC = 0.928) with 2 proteins (ALB, AFM), and distinguish DKD3 from DKD4 (AUC = 0.949) with 3 proteins (ANXA7, APOD, C9). Moreover, a 4-protein (SERPINA5, VPS4A, CP, TF) classifier was built to predict early stage DKD3 from diabetes (Fig. 4g). Importantly, a small-scale follow-up study on 11 patients showed that 2 high-risk pre-DKD patients predicted by our model had progressed to clinical DKD3, confirming the ability of our approach as a noninvasive method to monitor progression of renal dysfunctions, particularly for patients who have been classified as high-risk.

Previous studies showed that inactivation of similar biological processes may contribute to the disease progression [28, 29]. Samples in our dataset allowed us to analyze the different stages of the disease in a quantitative manner. We found that similar biological processes are indeed altered at different stages. For example, activation of complement cascades is unique for DKD4 and CKD, consistent with a recent study showing that higher urinary abundance of CFAH is associated with greater risk of progression to ESRD [30]. While activation of clotting cascade is enriched in both DKD and CKD (Figs. 2e and 1c), it is enriched with much higher statistical significance in CKD when the 3 datasets are compared, suggesting that the increase in coagulation factors in urine has the potential to distinguish CKD from DKD once these findings are validated by more experimental and clinical evidence.

Abnormal urine albumin excretion is a hallmark for incipient nephropathy. This has been attributed to dysfunction in glomerular permselectivity and tubular reabsorption, among other causes. Our data showed that, besides albumin, which is the 2nd most abundant urinary excreted proteins identified in our assay, other small serum proteins also showed an increase in excretion, consistent with the notion of renal dysfunction as an underlying cause. Recently, Van and colleague reviewed published DKD urinary proteomic/peptidomic literatures, and compiled a list of 75 most robust candidate markers at each stage of diabetic kidney disease and highlighted their roles in biological processes that may contribute to progression [31]. Of the 75 biomarkers, 55 (73.3%) can be detected in our dataset and 23 of them (41.8%) were among the top 100 abundant proteins in our dataset (Additional file 3: Table S1). Interestingly, many small serum proteins, including SERPINA1, TF, AMBP, APOA1, that showed increased excretion were expressed specifically or enriched in liver and bone marrow. On the other hand, proteins that showed decreased excretion, such as AHSG, CUBN, MASP2, showed low tissue specificity and were highly expressed in more diverse tissues. Notably, expressions of several decreased proteins have been reported in the literatures or Human Protein Atlas as enriched or specific in the tubular segment and play important functional roles in endocytosis.

Recent technical advancement in mass spectrometry empowered high throughput unbiased biomarker discoveries. For example, a urinary peptide-based classifier consisting of 273 naturally occurring urinary peptides was obtained from 3600 individuals analyzed by capillary electrophoresis coupled to MS. The so-called “CKD273” first proposed by Good et al. [32] can differentiate patients of chronical kidney diseases with different etiology including those caused by diabetes. Recently, the CKD273 classifier was applied in a trial for early detection of DKD (PRIORITY). This multicenter, prospective, observational study showed that a high-risk CKD273 score was associated with an increased risk of progression to microalbuminuria over a median of 2.5 years, independent of clinical characteristics. However, since the CE-MS detects naturally occurring urinary peptides without enrichment, the variety of proteins detected is often limited. In fact, the CKD273 peptides were derived from a total of 30 proteins and the collagen fragments constitute more than 66% of the 273 peptides. This low protein diversity provides limited biological insights into disease etiology and offered few treatment recommendations. Alternatively, analysis of urinary proteins, particularly with the enrichment of excreted vesicles, allows the detection and quantification of thousands of proteins from 5 ml of a urine sample, and the streamlined workflow also enables improved quantitative measurement.

Interestingly, collagens were not detected in high abundance as excreted proteins in our dataset. This is in contrast to results from urine peptidomics analyses represented by the CKD 273 panel, as Collagen a-1 (I) (126 fragments) and (III) (55 fragments) chains constitute > 66% of the 273 markers. Since the CKD273 detects naturally occurring peptides generated by endogenous proteases, these small proteins or peptides were likely lost in our sedimentation process. Together, these results suggest that proteomics and peptidomics analyses are complementary assays that could provide more accurate diagnosis with biological insights.

We acknowledge that a limitation of our study is the small sample size. A multi-center study is currently underway to enroll more patients so that the identified biomarkers can be validated in independent cohorts. Moreover, our results on DKD and CKD need to be interpreted with caution, as the CKD cases in our dataset have complex etiology. Larger sample size with more detailed clinical information combined with careful data analysis could reveal the underlying mechanism of the changes.

In summary, the urinary proteomic cross-sectional study allowed the differentiation of uncomplicated diabetes and DKDs at different stages, and the identification of biomarkers. Disease progression of the high-risk patients identified by the pre-DKD classifier was partly validated in a small-scale follow-up study. These results warrant the design of perspective studies to test the predictive power of our model for monitoring high-risk patients.

## Conclusions

In this study, we have established a urinary proteomic workflow to conduct a cross-sectional investigation of uncomplicated diabetes, DKD, and CKD patients. We identified biomarkers that could distinguish DKD from uncomplicated diabetes, and stage 4 DKD from stage 3 DKD. Logistic regression models were built upon the discovery dataset, and the classification was validated in an independent dataset. Bioinformatics analysis suggests that (upregulation of DKD4) complement cascade is an indication of DKD progression. Moreover, an algorithm was established to identify earlier stage DKD patients. Follow-up studies on 11 patients indicated that 2 putative pre-DKD patients have progressed to DKD3.

This study demonstrated the potential for urine proteomics as a noninvasive method for DKD diagnosis and identifying high-risk patients for progression monitoring. The pathways identified from differentially excreted proteins provided important clues on biological basis during disease progression.

## Supplementary Information

**Additional file 1: Figure S1.** A brief summary of proteomics analysis of human urine proteome. a. Pearson correlation coefficients of representative LC-MS/MS analyses of 293T cells as quality control samples. b. The dynamic range of urine protein abundance of high analytical confidence proteins. c. Number of GPs quantified in each urinary sample. d. Scatter plots showing the negative correlation between the three significant urinary proteins (RBP4, C7, ALB) and glomerular filtration rate.

**Additional file 2: Figure S2.** Risk score for predicting potential DKD patients based on the 4-protein classifier. a. Risk scores and the 4 marker protein expression levels of the high-risk “pre-DKD” patients at indicated times. The risk score was calculated by the predict_proba function in the sklearn (version 0.21.2) package based on Logistic Regression Classifier. b. Risk scores and the 4 marker protein expression levels of the diabetic patients.

**Additional file 3: Table S1.** Data information of 239 samples. Sheet 1: a list of 2946 proteins and their relative abundances (iFOT). Sheet 2: Clinical information of the 239 samples. Sheet 3: Biomarkers discovered in this study and those reported by Van et al. (2017) in reference 31.

**Additional file 4: Table S2.** Urine proteomics analysis of diabetes DKD, and CKD. Sheet 1: differentially excreted proteins between diabetes, DKD and CKD. Sheet 2: correlation analysis for the 2946 proteins and 13 routinely tested clinical or health indexes.

**Additional file 5: Table S3.** Differentially excreted proteins of diabetes and DKD. Sheet 1: differentially excreted proteins between diabetes and DKD in the discovery dataset (Batch 1). Sheet 2: differentially excreted proteins between diabetes and DKD in the validation dataset (Batch 2).

**Additional file 6: Table S4.** Differentially excreted proteins between DKD stage 3 and DKD stage 4. Sheet 1: differentially excreted proteins between DKD stage 3 and DKD stage 4 in Batch 1. Sheet 2: differentially excreted proteins between DKD stage 3 and DKD stage 4 in Batch 2.

**Additional file 7: Table S5.** Differentially excreted proteins of diabetes and DKD stage 3. Sheet 1: differentially excreted proteins between diabetes and DKD stage 3 in Batch 1. Sheet 2: differentially excreted proteins between diabetes and DKD stage 3 in Batch 2.

**Additional file 8: Table S6.** Differentially excreted proteins of diabetes and pre-DKD3.

**Additional file 9: Table S7.** Data information of the 13 follow-up patients. Sheet1: Sample information of the 13 patients. Sheet2: a list of 2549 proteins and their relative abundances (iFOT).  Sheet3: clinical information of the samples.

## Data Availability

The MS raw data generated in this study have been submitted to ProteomeXchange database (www.proteomexchange.org) via the iProX partner repository [33] under Accession Number IPX0002858000.
