# Supplementary figures and images for: Urine proteomics identifies biomarkers for diabetic kidney disease at different stages
Source: Clin Proteomics. 2021 Dec 29;18:32. doi: 10.1186/s12014-021-09338-6 (PMC8903606; doi:10.1186/s12014-021-09338-6)

# Supplemental figure 1

a

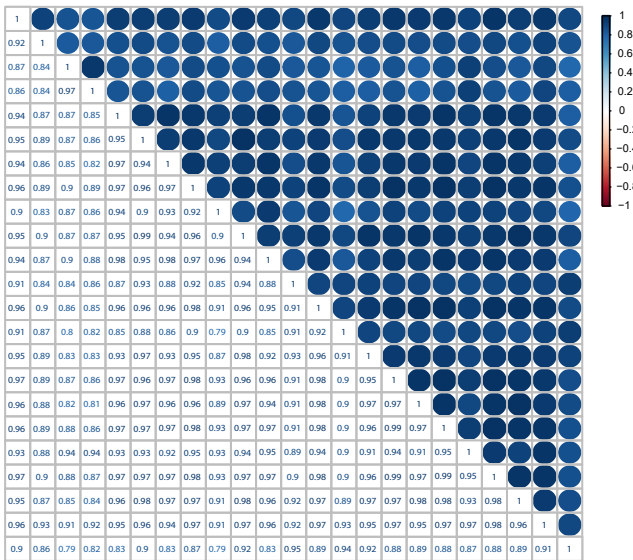

b

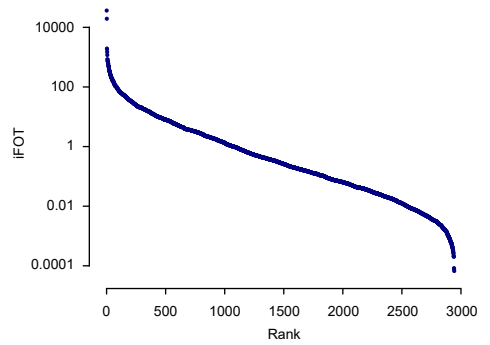

c

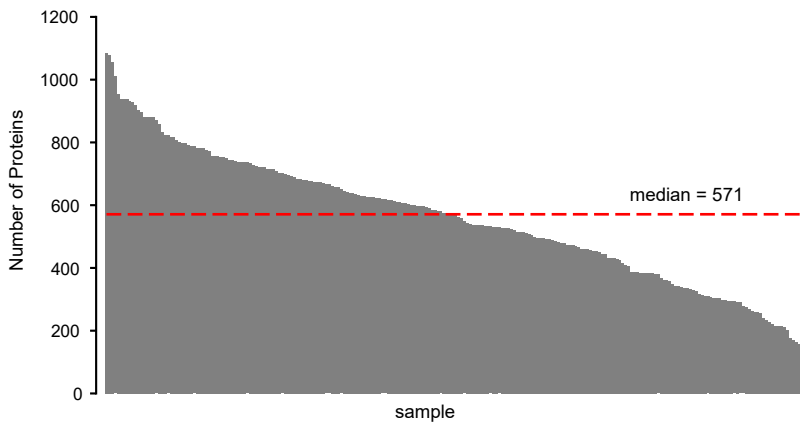

d

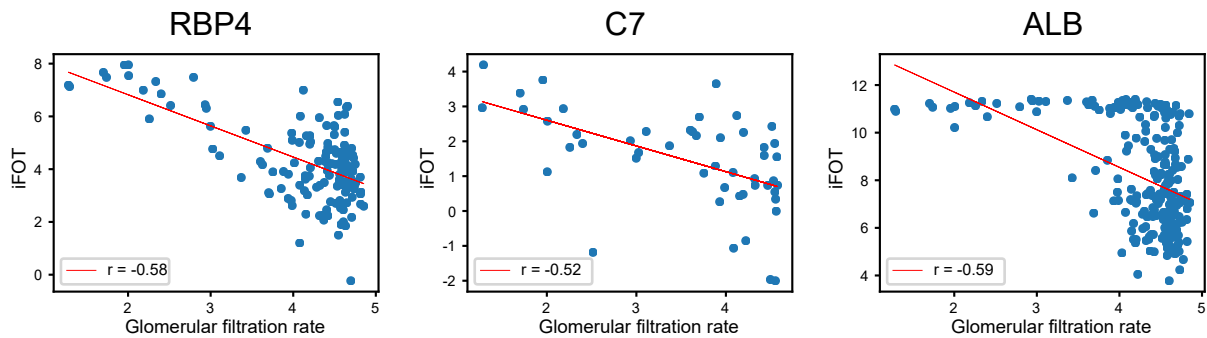

Supplement: Supplementary file 1 — Additional file 1: Figure S1. A brief summary of proteomics analysis of human urine proteome. a. Pearson correlation coefficients of representative LC-MS/MS analyses of 293T cells as quality control samples. b. The dynamic range of urine protein abundance of high analytical confidence proteins. c. Number of GPs quantified in each urinary sample. d. Scatter plots showing the negative correlation between the three significant urinary proteins (RBP4, C7, ALB) and glomerular filtration rate. [file 12014_2021_9338_MOESM1_ESM.pdf]

# Supplemental figure 2

a

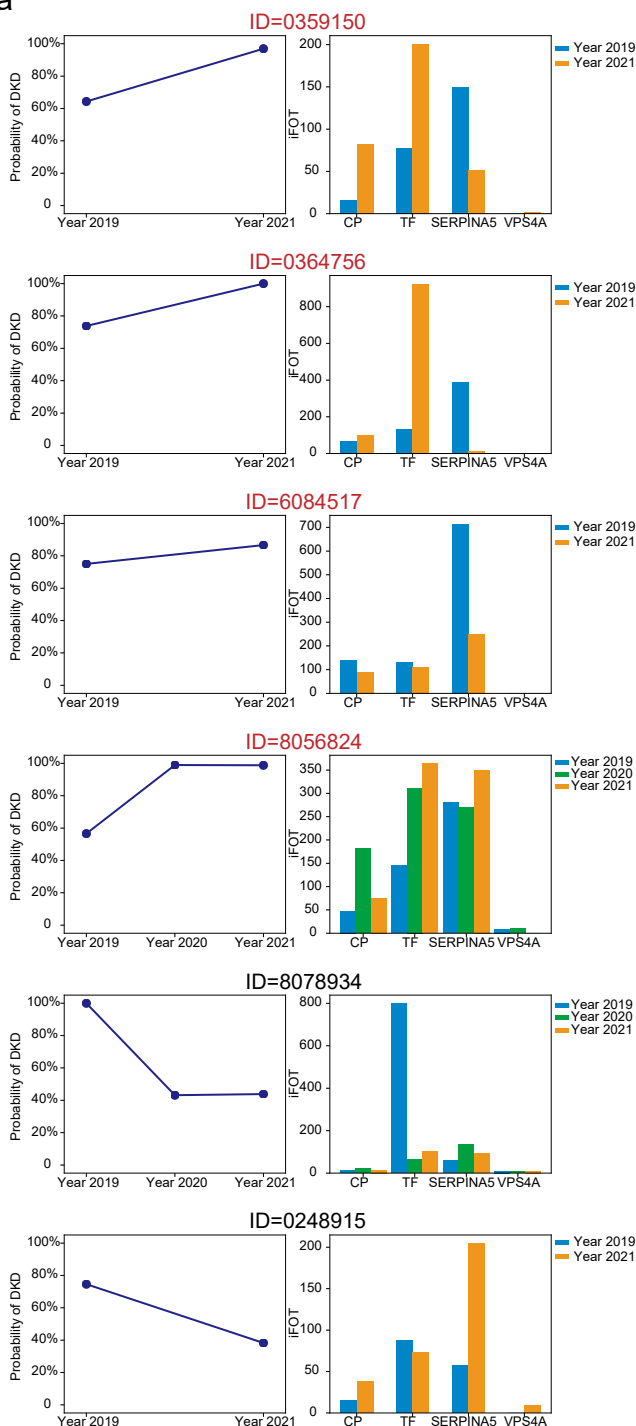

b

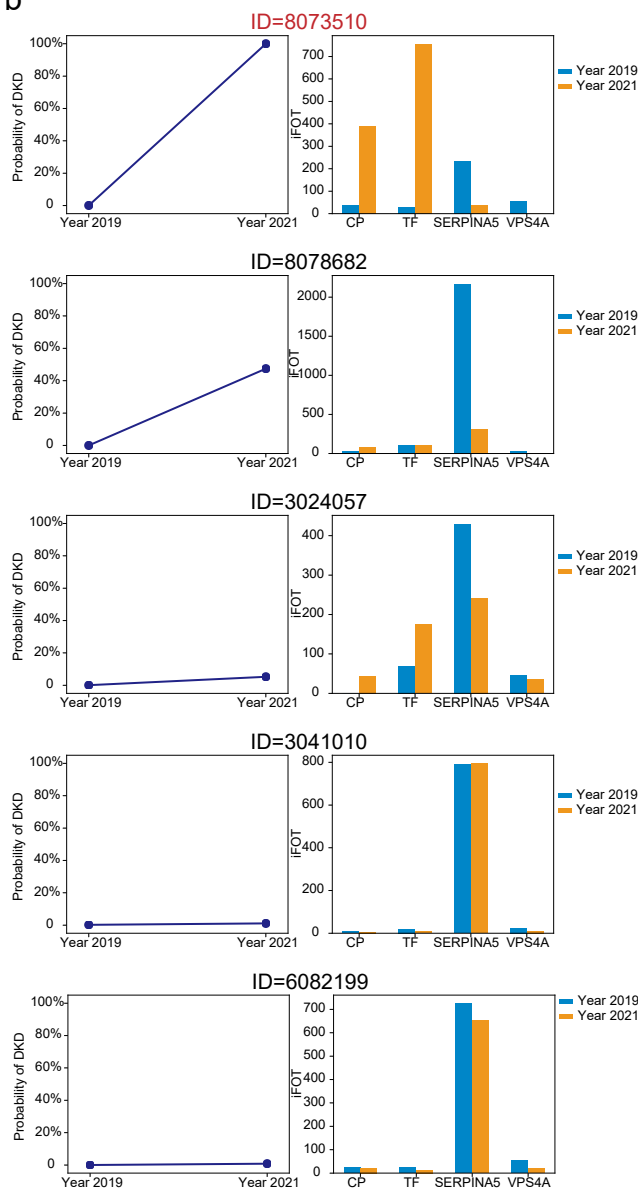

Supplement: Supplementary file 2 — Additional file 2: Figure S2. Risk score for predicting potential DKD patients based on the 4-protein classifier. a. Risk scores and the 4 marker protein expression levels of the high-risk “pre-DKD” patients at indicated times. The risk score was calculated by the predict_proba function in the sklearn (version 0.21.2) package based on Logistic Regression Classifier. b. Risk scores and the 4 marker protein expression levels of the diabetic patients. [file 12014_2021_9338_MOESM2_ESM.pdf]
